# The new perspective of cardiac exercise rehabilitation: based on integrative physiology

**DOI:** 10.3389/fphys.2025.1651589

**Published:** 2025-08-21

**Authors:** Guopeng You, Jinwen Xie, Wei Tong, Shaocong Zhao

**Affiliations:** ^1^ Department of Physical Education, Xiamen University of Technology, Xiamen, China; ^2^ Department of Physical Education, Changzhi Medical College, Changzhi, China

**Keywords:** cardiac rehabilitation, integrative physiology, exercise rehabilitation, cardiovascular, aerobic exercise

## Abstract

Cardiovascular diseases (CVDs) are the world’s leading cause of death, but there’s a gap between scientific research and real-world treatment. Exercise is a safe and effective way to prevent and manage CVDs, yet putting it into practice faces many challenges. This review shows how exercise protects the heart by improving metabolism, reducing inflammation and cell damage, and strengthening connections between heart cells and blood vessels. Exercise establishes a multi-organ defense network involving remote organs including the brain, skeletal muscle, adipose tissue, liver, and kidneys. To bridge the gap between research and clinical use, future efforts should focus on developing exercise-like drugs, personalized workout plans, and remote rehabilitation programs.

## 1 Introduction

Driven by global population aging and the widespread prevalence of risk factors, cardiovascular diseases (CVD) continue to exhibit rising incidence and mortality rates (Krishnan et al., 2025). Recent epidemiological studies reveal that while age-standardized CVD mortality decreased by 18.6% compared to 1990, over 20.5 million CVD-related deaths occurred globally in 2021, with ischemic heart disease (IHD) accounting for 48.3% of cases—a 72% absolute increase since 1990 (Martin et al., 2024). Data from the 2023 Report on Cardiovascular Health and Diseases in China underscore CVD as the leading cause of death nationwide, with coronary heart disease mortality demonstrating a persistent upward trajectory over the past decade (Center For Cardiovascular Diseases The Writing Committee Of The ReportOn Cardiovascular and Diseases In China, 2024). These findings highlight the urgent public health challenge posed by CVD.

Exercise intervention stands as a cornerstone strategy for CVD prevention and management. Clinical evidence confirms that regular, moderate physical activity significantly reduces CVD morbidity and mortality (Tucker et al., 2022). Animal experiments also shows that exercise improve the cardiac function of mice with myocardial infarction by inhibiting inflammation, oxidative stress and apoptosis (Bo et al., 2024; Wang et al., 2024; Hou et al., 2019). However, the *2023 WHO Global Report on Physical Activity* indicates that 27.5% of adults fail to meet recommended exercise guidelines (150–300 min of moderate or 75–150 min of vigorous activity weekly) (Bull et al., 2020). This disparity between scientific evidence and clinical implementation reflects systemic barriers to cardiac exercise rehabilitation, including inadequate insurance coverage for long-term exercise prescriptions (only 31% of U.S. insurance plans include cardiac rehabilitation), patient misconceptions regarding exercise safety, and a critical shortage of specialized rehabilitation teams (Balady et al., 2011; Schopfer et al., 2020). Addressing these challenges requires a deeper understanding of the systemic biological mechanisms underlying exercise-induced cardioprotection and the development of translational pathways bridging basic research to clinical practice. This study, based on the perspective of integrative physiology, systematically sorts out the mechanism by which the heart benefits from exercise, promoting the integration of basic research and clinical application, and has significant theoretical and practical significance.

## 2 Molecular regulatory mechanisms of exercise-induced cardiac functional improvement

### 2.1 Metabolic reprogramming

The heart, as a high-energy-demand organ, relies on metabolic homeostasis to maintain functional integrity. Under physiological conditions, 60%–90% of adenosine triphosphate (ATP) in adult cardiomyocytes is derived from fatty acid β-oxidation, while glucose metabolism contributes 10%–30%, with alternative substrates such as ketones and lactate becoming critical under specific physiological or pathological conditions (Lopaschuk et al., 2021; Kolwicz et al., 2013). Mitochondria, the primary ATP producers in cardiomyocytes, also serve as major sources of reactive oxygen species (ROS). Dysregulated mitochondrial dynamics contribute to myocardial injury and disease progression across multiple pathological models (Forte et al., 2021). The heart exhibits remarkable metabolic plasticity, dynamically adjusting substrate preferences in response to physiological demands or pathological states. Exercise-induced physiological remodeling is often accompanied by enhanced mitochondrial function. Long-term regular exercise can increase myocardial oxygen consumption by 3–10-fold, which elevates ADP concentration to enhance oxidative phosphorylation efficiency, ultimately leading to adaptive alterations characterized by enlarged mitochondrial volume and increased cristae density (Ritterhoff and Tian, 2023; Qiu et al., 2022; Abel and Doenst, 2011). This metabolic remodeling is closely associated with exercise modality and duration, with its molecular basis involving AMPK-PGC-1α signaling axis-mediated regulation of mitochondrial biogenesis (Qiu et al., 2022; Abel and Doenst, 2011; Li et al., 2018; White et al., 1987). Collectively, exercise training not only promotes metabolic adaptation toward higher efficiency in healthy hearts but also rehabilitates impaired myocardial mitochondrial energy production capacity and efficiency, thereby facilitating functional recovery in diseased hearts.

### 2.2 Regulation of oxidative stress

The core pathological mechanism underlying myocardial oxidative injury stems from disrupted redox homeostasis of ROS. Under physiological conditions, approximately 90% of ROS (e.g., superoxide anion O_2_•^-^ and hydrogen peroxide H_2_O_2_) derive from the mitochondrial electron transport chain (ETC), with the remainder originating from enzymatic systems such as NADPH oxidase (NOX) and xanthine oxidase (Zhou and Tian, 2018). In pathological states (e.g., ischemia-reperfusion injury, hypertensive cardiac hypertrophy) or during aging, mitochondrial complex I/III dysfunction amplifies electron leakage from the, ETC, coupled with upregulated NOX2/4 activity. These synergistic effects drive ROS production rates that substantially exceed the scavenging capacity of endogenous antioxidant systems (e.g., superoxide dismutase SOD and glutathione peroxidase GPx), culminating in oxidative overload (Burgoyne et al., 2012). This redox imbalance triggers lipid peroxidation (e.g., elevated malondialdehyde MDA), protein carbonylation (e.g., 3-nitrotyrosine accumulation), and mitochondrial DNA oxidative damage (e.g., increased 8-hydroxy-2′-deoxyguanosine 8-OHdG) via Fenton reactions, ultimately promoting cardiomyocyte apoptosis and contractile dysfunction (Tsutsui et al., 2011).

Distinct exercise modalities differentially regulate myocardial antioxidant systems. Moderate-intensity continuous training (MICT) significantly enhances SOD1/2 activity and the GSH/GSSG (Glutathione and glutathione disulfide) ratio, mediated by exercise-induced Sirt3 (Sirtuin 3) deacetyation (Sundaresan et al., 2009). High-intensity interval training activates the HIF-1α/Nrf2 (Hypoxia-inducible factor 1α/Nuclear factor erythroid 2-like 2) synergistic pathway through transient hypoxia, substantially improving Nrf2 nuclear translocation efficiency (Gliemann et al., 2016). Resistance exercise promotes FoxO transcription factor phosphorylation via IGF-1/Akt signaling, driving SOD2 and CAT gene expression, though with minimal effects on GPx regulation (Konopka and Harber, 2014). A randomized controlled trial in coronary artery disease patients revealed that 12-week aerobic exercise (150 min/week, it is the minimum recommended by the ACSM in reference to exercise) increased Smyd1 (SET And MYND Domain Containing 1) expression by 1.8-fold in myocardial biopsy samples, accompanied by a 37% reduction in plasma malondialdehyde (MDA) levels and a 4.2% improvement in left ventricular ejection fraction (LVEF) (Hambrecht et al., 1993).

### 2.3 Regulation of programmed cell death

Programmed cell death (PCD) in cardiomyocytes—encompassing apoptosis, ferroptosis, and pyroptosis—constitutes a critical regulatory mechanism for maintaining cardiac homeostasis through distinct molecular cascades. Apoptosis, a caspase-dependent process, physiologically eliminates senescent or damaged cardiomyocytes but is pathologically activated by stimuli that elevate the Bax/Bcl-2 ratio, triggering mitochondrial permeability transition pore (mPTP) opening, cytochrome C release, and caspase-3 activation, ultimately causing irreversible contractile unit loss (Krijnen et al., 2002). Ferroptosis, an iron-dependent, lipid peroxidation-driven death modality, involves glutathione peroxidase 4 (GPX4) inactivation and mitochondrial cristae collapse (Stockwell et al., 2017). Disrupted myocardial iron homeostasis (e.g., free iron overload) catalyzes polyunsaturated fatty acid (PUFA) peroxidation via the Fenton reaction, compromising plasma membrane integrity. Exercise preconditioning suppresses ferroptosis in doxorubicin-induced cardiotoxicity by activating mitochondrial superoxide-dependent AMPKα2, which inhibits the p53-SLC7A11 axis and enhances ROS scavenging, concurrently downregulating ferroptosis markers (ACSL4, PTGS2) (Tadokoro et al., 2020). Chronic exercise further bolsters antioxidant defenses via the Nrf2/GPX4 pathway (Wang et al., 2022a). Pyroptosis, a caspase-1-dependent inflammatory death initiated by NLRP3 (NOD-like receptor family, pyrin domain containing-3) inflammasome activation, features gasdermin D (GSDMD)-mediated pore formation and IL-1β release (Jankowska et al., 2008). The ROS-NLRP3-IL-18 axis amplifies inflammation during myocardial ischemia-reperfusion injury, impairing contractility (Gopalan et al., 2021). Combined curcumin and exercise intervention in hyperlipidemic rats downregulates pyroptosis genes (NLRP3, ASC (Apoptosis-associated speck-like protein containing a CARD), caspase-1) by inhibiting TLR4/MyD88/NF-κB (Toll-like receptor 4/Myeloid differentiation factor 88/Nuclear factor kappa-B) signaling and reducing serum IL-1β (Ramos et al., 2016). While exercise alone partially mitigates pyroptosis (Yang et al., 2025), its direct mechanisms (e.g., inflammasome regulation) require validation via conditional knockout models.

### 2.4 Epigenetic regulation

Epigenetic modifications—including dynamic alterations in DNA methylation, non-coding RNAs, and histone modifications—orchestrate exercise-induced cardioprotection by remodeling myocardial gene expression in response to pathological stress. Exercise modulates cardiac DNA methylation through regulation of DNMTs and TET demethylases (Benito et al., 2021; Haeusler et al., 2013). In hypertensive cardiac hypertrophy, hypomethylation of the ACE promoter elevates ACE mRNA expression, augmenting angiotensin II (Ang II)-mediated fibrosis (Benito et al., 2021); conversely, 12-week aerobic exercise increases ACE promoter methylation and reduces plasma Ang II (Haeusler et al., 2013). Furthermore, aerobic exercise ameliorates myocardial ischemia-reperfusion injury by suppressing METTL3-mediated m6A methylation, thereby stabilizing cell death-related mRNAs (Zhang et al., 2025). MiRNA is currently regarded as a potential therapeutic target and biomarker in the study of various physiological and pathological processes in cardiovascular diseases (van Rooij and Olson, 2007). Exercise also remodels myocardial miRNA profiles (Soci et al., 2011). Aerobic training increases the expression of miR-29, reduces the expression and concentration of collagen genes in the heart, and promotes physiological myocardial hypertrophy (Soci et al., 2011). Where in upregulated miR-29b directly targets collagen genes (COL1A1/COL3A1/ELN) to attenuate post-infarction fibrosis (Melo et al., 2014), while exercise-induced HIF-1α activates miR-126, promoting angiogenesis via PI3K/AKT/eNOS and MAPK pathways in infarcted hearts (Song et al., 2020). Intermittent aerobic exercise can inhibit the TGFβ pathway by up-regulating the expression of miR-101a, ultimately leading to a reduction in cardiac tissue fibrosis and scar formation (Xiao et al., 2017). Systemically, exercise-stimulated skeletal muscle releases exosomal miR-126-3p that suppresses cardiomyocyte VCAM-1 expression, mitigating endothelial inflammation (Bei et al., 2017). In addition, physical exercise is increasingly recognized for its ability to regulate cardiac function by regulating histone modifications (Zheng et al., 2025). Lehmann et al. found that compared with healthy controls, failing hearts showed reduced levels of HDAC4 N-terminal fragment (HDAC4-NT), and exercise was proven to increase HDAC4-NT levels to protect cardiac function (Lehmann et al., 2018). Meanwhile, during exercise, AMPK in the myocardium is activated and phosphorylates HDAC4. This phosphorylation reduces the inhibitory effect of HDAC4 on MEF2a, and this change helps improve cardiac function and glucose metabolism in mice with heart failure (Jiang et al., 2020).

## 3 Cellular crosstalk

### 3.1 Cardiomyocyte-endothelial dialogue

Cardiomyocytes and cardiac microvascular endothelial cells (CMECs) form dynamic functional units through paracrine signaling, mechanical coupling, and metabolic interactions, collectively maintaining cardiac homeostasis. This bidirectional communication (cardiomyocyte-endothelial dialogue) critically regulates energy metabolism, redox balance, and pathological remodeling.

Nitric oxide (NO) suppresses excessive L-type calcium channel activation in cardiomyocytes via the cGMP/PKG pathway, reducing diastolic Ca^2+^ concentration and alleviating calcium overload-induced systolic dysfunction (Ramos et al., 2016). Pathological conditions (e.g., hypertension) induce endothelial endothelin-1 (ET-1) overexpression, which activates the cardiomyocyte CaMKII/NFATc3 pathway through ETA receptors, driving pathological hypertrophy (Al-Khatib et al., 2018).

Exercise enhances cardiomyocyte-endothelial communication by improving paracrine signaling. Exercise upregulates NRG-1 expression in endothelial progenitor cells (EPCs). Post-myocardial infarction exercise increases eNOS expression and promotes angiogenesis through the NRG-1/ErbB4/PI3K/AKT signaling pathway (Huang et al., 2025). Additionally, the mechanisms by which exercise strengthens cardiomyocyte-endothelial communication involve enhanced mechanical coupling. Exercise-induced cyclic shear stress upregulates endothelial Piezo1 expression and inhibits ET-1 production via AMPKα activation (Clarkson et al., 1999). In conclusion, exercise improves cardiac function by reinforcing cardiac-endothelial communication, promoting endothelial proliferation, and enhancing angiogenesis.

### 3.2 Immune cell regulation

Immune cells serve as pivotal regulators of cardiac homeostasis and disease progression through dynamic orchestration of inflammatory responses and repair processes. During acute myocardial injury, vascular endothelial upregulation of adhesion molecules and resident macrophage-derived chemokines recruit neutrophils and monocytes for necrotic clearance and inflammation resolution. Subacutely, amplified pro-inflammatory signaling induces mast cell release of pro-fibrotic factors, driving maladaptive fibrosis and dysfunction. Exercise exerts potent immunomodulatory effects by mobilizing circulatory lymphocytes/neutrophils and reprogramming cardiac immunity: (1) Post-ischemic DAMP-activated TLR4/MyD88/NF-κB signaling promotes M1 macrophage polarization and pro-apoptotic cytokine secretion (IL-1β, TNF-α) (Nahrendorf and Swirski, 2016), which exercise counteracts via PPARα-mediated inhibition of NF-κB nuclear translocation to reduce M1 dominance (Santos et al., 2016); (2) Exercise-induced IL-4/IL-13 activates STAT6 to drive M2 macrophage polarization, enhancing TGF-β1/VEGF-A-dependent collagen remodeling and angiogenesis (Lavine et al., 2014). Single-cell sequencing confirms significant enrichment of pro-repair genes (*Arg1*, *Ym1*) in cardiac macrophages of exercised subjects (Epelman et al., 2014). Collectively, exercise mitigates pathological remodeling by suppressing excessive immune activation while promoting macrophage phenotypic switching toward reparative M2 polarization.

## 4 Exercise-mediated organ dialogue

**FIGURE 1:**
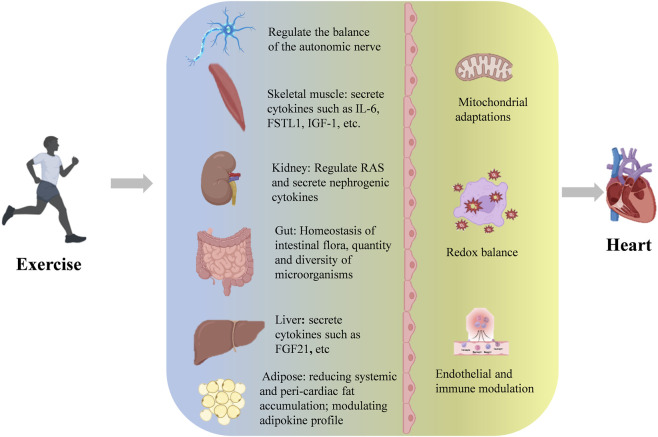
The multi-layered mechanisms by which exercise confers cardioprotection.

Exercise has been substantiated as an effective approach for primary prevention and adjunctive therapy of cardiovascular diseases, with its clinical value strongly supported by evidence-based medicine. Recent breakthroughs in molecular mechanism studies further elucidate its therapeutic potential. From a systems biology perspective, exercise intervention establishes multidimensional regulatory networks that induce cascade adaptive changes spanning subcellular structures to tissue and organ levels. Importantly, these biological effects exhibit remarkable inter-organ crosstalk. Mechanistic investigations reveal that exercise-mediated cardioprotection operates through multiple organ axes, including but not limited to the brain-heart axis, skeletal muscle-heart axis, liver-heart axis, adipose-heart axis, kidney-heart axis, and gut-heart axis (Figure 1).

### 4.1 Autonomic nerve remodeling

#### 4.1.1 Autonomic balance regulation

Cardiac autonomic regulation—sympathetic and parasympathetic balance—critically governs myocardial function, with its dysfunction being a primary driver of cardiac injury and heart failure (Floras, 2009). Pathological states (e.g., pressure overload) trigger hypothalamic paraventricular nucleus (PVN) glutamatergic neuron hyperexcitability, leading to chronic sympathetic overactivation characterized by excessive norepinephrine (NE) release and impaired reuptake (Florea and Cohn, 2014). While acutely compensatory, sustained NE excess exacerbates myocardial damage via β1-AR/cAMP/PKA-induced calcium overload and mitochondrial oxidative stress, accelerating ventricular remodeling (Floras, 2009). Exercise restores autonomic homeostasis through multi-tiered mechanisms: (1) Downregulating PVN angiotensin II type 1 receptor (AT1R) to suppress glutamatergic hyperactivity, improving ischemic cardiac function (Patel and Zheng, 2012); (2) Enhancing baroreceptor sensitivity while reducing β2-adrenergic receptor responsiveness to catecholamines (Fraga et al., 2007); (3) Restoring the adrenal GRK2-α2-AR-catecholamine axis to normalize sympathetic tone (Rengo et al., 2010). Conversely, impaired vagal activity reduces heart rate variability (HRV)—a biomarker of cardiac autonomic integrity (Billman et al., 2015)—which correlates with myocardial infarction and heart failure risk (Sessa et al., 2018; Hillebrand et al., 2013). Regular exercise elevates cardiac vagal tone, concurrently attenuating sympathetic activity and β2-AR sensitivity, thereby augmenting HRV and conferring cardioprotection in both healthy and diseased hearts (Routledge et al., 2010; Fiuza-Luces et al., 2018). In conclusion, exercise preserves and restores cardiac autonomic homeostasis, modulates HRV, and thereby confers cardioprotection.

#### 4.1.2 Optimization of cardiovascular reflex regulation

The neural regulation of cardiac activity involves three primary reflex pathways: baroreflex, chemoreflex, and volume reflex. The baroreceptor reflex monitors blood pressure changes through the carotid sinus and aortic arch baroreceptors. When blood pressure rises, it reduces heart rate and cardiac output by enhancing vagal nerve activity, constituting an important mechanism for maintaining blood pressure homeostasis. Hypertensive patients generally have reduced baroreceptor sensitivity, and regular exercise not only lowers blood pressure but also effectively restores baroreflex sensitivity (Brum et al., 2000; Laterza et al., 2007), which has significant clinical implications for improving cardiac load. The chemoreceptor reflex is mainly activated by the carotid body and aortic body chemoreceptors when blood oxygen partial pressure drops, carbon dioxide partial pressure rises, and pH decreases, enhancing sympathetic nerve activity to increase heart rate and cardiac output. Notably, in chronic heart failure, chemoreceptor reflex sensitivity abnormally increases, leading to excessive sympathetic nerve activation and accelerating myocardial remodeling (Sun et al., 1999). Animal experiments have confirmed that exercise intervention can effectively inhibit the sensitivity of peripheral chemoreceptors in heart failure models (Li et al., 2008), providing a mechanistic explanation for the improvement of cardiac function through exercise rehabilitation. The volume receptor reflex monitors the circulatory volume status through atrial stretch receptors and cardiopulmonary pressure receptors. When blood volume increases, it reduces sympathetic nerve tension and enhances vagal nerve activity by inhibiting the renin-angiotensin-aldosterone system and increasing atrial natriuretic peptide secretion, thereby reducing cardiac output. In pathological conditions, weakened myocardial contractility leads to increased residual blood volume in the ventricle, and abnormally activated volume reflex may form a vicious cycle of “increased preload - reflexive cardiac function inhibition.” Exercise effectively regulate preload and afterload by promoting blood redistribution in skeletal muscles and improving venous return, possibly interrupting this pathological process. Existing evidence indicates that the multi-target regulation of cardiovascular reflexes by exercise is an important mechanism for its cardioprotective effect. However, the coordinated action mechanism of the three reflex pathways during exercise intervention, their temporal regulatory characteristics, and their dynamic balance relationship under pathological conditions still require systematic research. In particular, the differences in the effects of different exercise modes (intensity, duration) on each reflex pathway and the central integration mechanism are worthy of in-depth exploration.

#### 4.1.3 Central neural remodeling mechanisms

The cardiac neural regulatory network exhibits multi-level characteristics, involving not only autonomic nerves and cardiovascular reflexes but also the “brain-heart” regulatory axis formed by the higher cortical-hypothalamic-brainstem pathways. Functional magnetic resonance imaging (fMRI) studies confirm that limbic systems (e.g., lateral prefrontal cortex, insular cortex, amygdala) form neural circuit connections with the heart via the hypothalamic PVN (Hu et al., 2023). Notably, the primary motor cortex (M1 region), traditionally associated with motor execution and cognitive functions (Levy et al., 2020; Shenoy et al., 2013; Bhattacharjee et al., 2021), has recently been found to exhibit anatomical connectivity with the heart (Li et al., 2021), Optogenetic experiments demonstrate that activating M1 glutamatergic neurons bidirectionally modulates heart rate and contractile function in both healthy and myocardial infarction (MI) mice (Bo et al., 2024).

Myocardial infarction-induced neural remodeling involves dual central and peripheral pathological alterations. MI enhances sympathetic excitatory input from the PVN to the rostral ventrolateral medulla (RVLM), forming a hyperactive “PVN-RVLM-sympathetic nerve” axis that directly causes abnormal β-adrenergic receptor density elevation and myocardial fibrosis (Koba et al., 2020; Wang et al., 2022b). Exercise intervention effectively suppresses sympathetic sprouting and catecholamine secretion by downregulating NADPH oxidase activity and inhibiting oxidative stress in the RVLM, demonstrating clear cardioprotective effects in MI animal models (Koba et al., 2014; Chen et al., 2014). Current evidence reveals that exercise improves cardiac autonomic balance by modulating multi-level central nodes in the “cortex-hypothalamus-brainstem” axis. However, critical questions remain unresolved: (1) hierarchical regulatory relationships among distinct brain regions in exercise-mediated cardioprotection; (2) temporal window characteristics of neural plasticity; (3) dose-response relationships between exercise intensity and neural remodeling effects. Particularly, the regulatory roles of non-motor cortical regions (e.g., insula, anterior cingulate cortex) in the “brain-heart” axis warrant further investigation.

### 4.2 Skeletal muscle-cardiac crosstalk

As the largest metabolic organ, skeletal muscle exerts its endocrine function as a pivotal mediator in exercise-induced cardioprotection. During exercise, skeletal muscle secretes over 650 bioactive myokines (Bay and Pedersen, 2020), which establish bidirectional communication with multiple organs (e.g., brain, cardiovascular system) via endocrine pathways, forming the systemic biological basis of exercise benefits (Severinsen and Pedersen, 2020). Notably, clinical experiment have shown that skeletal muscle-derived IL-6 exhibits unique anti-inflammatory properties in exercise physiology: while circulating IL-6 levels rise markedly during exercise, it induces monocytes to produce anti-inflammatory mediators (e.g., IL-1 receptor antagonist, IL-10) while suppressing the bioactivity of pro-inflammatory cytokines like TNF-α, creating a systemic anti-inflammatory milieu (Fiuza-Luces et al., 2018; Steensberg et al., 2003; Starkie et al., 2003). Animal experiments revealed that exercise-induced follistatin-like protein 1 (FSTL1) improve cardiac function after myocardial infarction through a comprehensive protective effect of inhibiting apoptosis, reducing fibrosis and promoting angiogenesis (Ouchi et al., 2008; Xi et al., 2021). Myokines such as irisin and insulin-like growth factor 1 (IGF-1) show significant cardioprotective effects in animal models of cardiac ischemia-reperfusion injury and pressure overload by regulating myocardial energy metabolism, enhancing antioxidant capacity and improving mitochondrial function (Ma et al., 2021; Li et al., 2024; Tan et al., 2023). Current evidence confirms that exercise regulates multiple pathophysiological processes (cardiac inflammatory microenvironment, angiogenesis, cell survival) through myokine cascades along the skeletal muscle-heart axis. However, critical gaps remain: (1) spatiotemporal secretion patterns of distinct myokines; (2) tissue-specific receptor distribution; (3) crosstalk between signaling pathways; and particularly; (4) dose-response relationships between exercise intensity and myokine secretion profiles require systematic investigation.

### 4.3 Kidney-heart crosstalk

The cardiorenal interaction plays a pivotal role in cardiovascular pathophysiology, classically exemplified by cardiorenal syndrome (CRS), where dysfunction of either the heart or kidneys triggers secondary injury in the other organ through neurohumoral regulation, hemodynamic alterations, and inflammatory responses (Ronco et al., 2010). Epidemiological studies reveal a significantly elevated cardiovascular mortality rate in chronic kidney disease (CKD) patients, with global CKD-related cardiovascular deaths reaching 1.8 million in 2021 (Martin et al., 2024). Pathologically, CKD-induced cardiac injury arises from a triad of mechanisms: (1) increased left ventricular end-diastolic pressure due to volume overload; (2) enhanced oxidative stress from uremic toxin accumulation; and (3) overactivation of the renin-angiotensin-aldosterone system (RAAS) (Chuasuwan and Kellum, 2012). Exercise ameliorates cardiorenal interactions through multiple pathways. Regular exercise reduces intraglomerular pressure and suppresses tubulointerstitial fibrosis progression, thereby decelerating CKD advancement (Shlipak et al., 2022; Yamakoshi et al., 2022). Exercise-induced enhanced sodium excretion and modified antidiuretic hormone sensitivity alleviate volume overload (Beetham et al., 2022). Studies demonstrate that exercise stimulates renal synthesis of ELABELA (ELA), a dual-organ protective peptide hormone. ELA activates the YAP-Akt-mTOR-P70S6K signaling network in cardiomyocytes, enhancing contractile reserve, promoting microvascular angiogenesis, and suppressing Ang II-induced pathological remodeling to preserve cardiac function (Zheng et al., 2021; Xi et al., 2023). Current evidence confirms that exercise intervention establishes a multi-target protective network against CRS by: (1) maintaining glomerular filtration function; (2) modulating RAAS activity; and (3) augmenting renoprotective factor secretion. However, critical gaps persist regarding: (1) temporal effects of exercise on cardiorenal crosstalk; (2) differential cardiorenal benefits across exercise modalities (endurance/resistance/high-intensity interval training); and (3) roles of other renally derived cytokines beyond ELA in exercise-mediated cardioprotection.

### 4.4 Gut-heart cross-talk

The gut microbiota, as the largest exogenous metabolic organ, dynamically regulates systemic homeostasis through microbiota-host co-metabolic networks. Microbial metabolites serve as chemical mediators of gut-organ axis communication and play vital roles in maintaining cardiovascular health (Nicholson et al., 2005; Dai et al., 2023). Gut dysbiosis impairs cardiac function via: (1) systemic inflammation triggered by pathogen-associated molecular pattern (PAMP) translocation; (2) pro-atherogenic metabolite production (e.g., trimethylamine N-oxide, TMAO); and (3) cholesterol homeostasis disruption caused by bile acid metabolism dysregulation. Exercise exerts significant regulatory effects on gut microbiota. It optimizes microbial composition (Kim and Kang, 2019) and enhances microbiome richness/diversity (Clarke et al., 2014). Notably, myocardial ischemia itself alters gut microbiota diversity. Both human and animal studies demonstrate post-myocardial infarction microbiome shifts (Tang et al., 2019; Liu et al., 2017), while exercise increases Butyricimonas and Akkermansia abundance, improving cardiac function in infarcted hearts (Liu et al., 2017). Mechanistically, exercise-induced microbial metabolic reprogramming generates cardioprotective molecules: 3-hydroxypyridinecarboxylic acid (3-HPA) and 4-hydroxybenzoic acid (4-HBA) activate the Nrf2-ARE pathway, reducing cardiomyocyte apoptosis by 42% and suppressing TGF-β/Smad3-mediated collagen deposition, ultimately limiting infarct size (Zhou et al., 2022). These findings suggest exercise protects ischemic hearts through dual mechanisms—microbiota structural optimization and functional metabolite production. However, critical gaps remain regarding: (1) exercise intensity-metabolite concentration gradients; (2) causal contributions of specific bacterial strains; and (3) tissue-specific delivery mechanisms of microbial metabolites.

### 4.5 Liver-heart crosstalk

Hepato-cardiac interactions play significant roles in interorgan pathophysiological communication. Common liver diseases may induce cardiac dysfunction (Correale et al., 2018). Exercise intervention disrupts this vicious cycle through multiple mechanisms. Exercise alleviates cirrhosis-associated cardiac remodeling and diastolic dysfunction (de Souza et al., 2021). At the molecular mechanism level, the core mediators of heart-liver interaction include: (1) Inflammatory signal cascade (Correale et al., 2018):Myocardial infarction induces inflammatory responses in the liver and leads to liver injury, while exercise-induced myogenic factor Irisin inhibits liver inflammatory responses and improves liver injury caused by myocardial infarction (Wang et al., 2023a). (2) Hepatogenic inducible factor: Hepatogenic Protein coagulation Factor XI (FXI) can activate the Bone Morphogenetic Protein (BMP)-Smad1/5 pathway in the heart, thereby inhibiting the genes involved in inflammation and fibrosis and protecting the cardiac function in heart failure (Cao et al., 2022). FGF21 is a cytokine mainly expressed by the liver and also an exercise-inducing factor. Exercise can maintain mitochondrial integrity through the FGF21-Sirtuin3 axis to protect cardiac function under pathological conditions (Jin et al., 2022). Exercise bidirectionally regulates hepato-cardiac crosstalk, attenuating cardiac injury through interorgan anti-inflammatory effects and direct myocardial protection via hepatokines. In heart failure, the ratio of phosphocreatine to ATP in the myocardium significantly decreases, leading to insufficient energy supply to the heart (Jullig et al., 2008). Exercise can induce the expression of myocardial FGF21 coreceptor β-klotho, promote the phosphorylation of FOXO3 through AMPK signaling, induce the expression of mitochondrial deacetylase Sirt3, promote the deacetylation of myocardial mitochondrial enzyme clusters to maintain mitochondrial integrity and function, and improve the efficiency of mitochondrial oxidative phosphorylation (Jin et al., 2022). Restore myocardial ATP levels and improve impaired cardiac function. During the rational remodeling process of heart disease, the preference for energy metabolism substrates shifts from fatty acid oxidation to glucose oxidation, resulting in a decrease in energy productivity (Gibb and Hill, 2018). Exercise can promote the expression of medium-chain acyl-coA dehydrogenase and 2, 4-dienyl-CoA reductase one in mitochondria, enhance the β -oxidation capacity of myocardial fatty acids, and improve the energy supply efficiency of the heart (Risikesan et al., 2023). However, unresolved questions include: (1) tissue-specific FGF21 regulation (liver vs. adipose) in response to exercise; (2) time-dose relationships between exercise intensity and FGF21 effects; and (3) roles of exercise-induced hepatic metabolites (e.g., bile acid derivatives) in the liver-heart axis.

### 4.6 Adipose-heart crosstalk

Obesity and metabolic syndrome are major cardiovascular risk factors, with adipose tissue playing a central role. Obesity induces systemic inflammation (Ghigliotti et al., 2014; Berg and Scherer, 2005), which paradoxically drives adipogenesis as an adaptive mechanism to prevent ectopic fatty acid deposition (Wernstedt Asterholm et al., 2014). Epicardial adipose tissue exhibits heightened adipogenic sensitivity compared to other visceral fat depots (Marchington and Pond, 1990). In obesity, epicardial fat acts as a sensor, mediating systemic inflammatory effects on myocardium akin to its adverse coronary impacts (Packer, 2018). Exercise reduces epicardial fat accumulation, mitigates oxidative stress/inflammation, and confers cardioprotection (Nyawo et al., 2021). Adipokines exhibit context-dependent cardiac effects: Leptin protects against cardiomyocyte hypertrophy/apoptosis under physiological conditions (Unger, 2005), but promotes adverse cardiovascular outcomes in obesity-related hyperleptinemia (Zhao et al., 2021). Exercise lowers hyperleptinemia and restores leptin’s cardioprotective effects (Lowndes et al., 2014). In summary, exercise combats adipose-mediated cardiac injury by: (1) reducing systemic and peri-cardiac fat accumulation; and (2) modulating adipokine profiles. However, mechanistic details of exercise-regulated leptin signaling and tissue-specific adipokine interactions require further elucidation.

## 5 Clinical translation opportunities and challenges

### 5.1 Exercise mimetics

Recent years have witnessed growing interest in bioactive oral compounds that mimic or amplify exercise benefits, termed “exercise mimetics” or “exercise pills.” These compounds aim to stimulate muscle adaptations akin to exercise, yet their capacity to fully replicate exercise effects remains contentious. Scholars argue that exercise-induced responses involve multifactorial, often redundant interactions among signaling kinases, downstream pathways, and spatiotemporal coordination, generating integrated adaptations to physiological challenges (Hsieh et al., 2025). Consequently, single-target interventions are unlikely to recapitulate the full exercise phenotype. Nevertheless, key regulatory nodes retain therapeutic potential. Over 100 myokines have been identified (Chow et al., 2022), though most remain functionally uncharacterized. Beyond myokines, muscle-derived metabolites during contraction also contribute to metabolic regulation, suggesting “exercise mimetics” may partially mimic metabolic benefits while neglecting multisystem adaptations (Hsieh et al., 2025; Hoffmann and Weigert, 2017). Future research should employ multi-omics technologies to delineate cardioprotective exercise factors, establish drug screening platforms based on exercise factor interactions, and develop targeted delivery systems for exercise-limited patients. Breakthroughs in these areas may bridge basic discoveries to clinical translation, advancing exercise mimetics from concept to application.

### 5.2 Personalized exercise prescription

Scientific exercise prescription adheres to the FITT-VP framework (Frequency, Intensity, Time, Type, Volume, Progression), which synergistically modulates energy metabolism, hemodynamics, and molecular signaling to determine pathophysiological outcomes. Given the stringent safety and efficacy requirements for cardiac exercise rehabilitation, careful consideration must be given to differential cardiac impacts associated with varying exercise parameters. Pandey et al. believe that there is a clear dose-response relationship between exercise and cardiovascular health benefits (Pandey et al., 2015). Zheng et al. demonstrated that individuals engaging in moderate and large exercise volumes exhibited superior cardiac structural parameters compared to those performing high-intensity exercise (Zheng et al., 2024). Zhang et al. found that the greatest benefits were gained when exercise was initiated in the acute phase after myocardial infarction, while the later the exercise intervention after myocardial infarction, the worse the exercise effect (Zhang et al., 2016). It is noteworthy that High-Intensity Interval Training (HIIT) demonstrates superior efficacy in suppressing pathological cardiac remodeling in patients with heart failure compared to aerobic exercise, resistance training, and combined training modalities (Wang et al., 2023b; Cornelis et al., 2016). Meanwhile, a higher training frequency (for example, more than twice a week) can better improve the endothelial function of patients with heart failure than a lower training frequency (for example, twice a week) (Fuertes-Kenneally et al., 2023). Pandey et al. observed that when the PA (Physical Activity) levels were 250 and 500 MET-min/wk, the risk of HF decreased by only 5% and 10%, respectively. However, individuals who engaged in physical activity at 1000 MET-min/wk and 4 times 2000 MET-min/wk had a 19% and 35% reduced risk of heart failure (Pandey et al., 2015). From this, it can be seen that exercise parameters are of vital importance to the effect of cardiac exercise rehabilitation. Although some basic research has compared the differences in the protective effects of different exercise parameters on the heart, in clinical practice, patients’ conditions, disease courses, and physical constitutions all vary. Therefore, the setting of exercise parameters needs to be considered appropriately based on the actual situation (Pei et al., 2023; Pei et al., 2021; Zhang et al., 2024; Sylviana et al., 2022). Future work should establish multidimensional parameter matrices encompassing exercise modalities and intervention windows, integrated with single-cell sequencing and metabolomics to map exercise parameter-molecular network-clinical outcome pathways. This paradigm may transcend empirical prescription, enabling precision cardiac rehabilitation.

Although exercise is regarded as a safe and effective remedy for preventing and treating cardiovascular diseases, its potential risks, contraindications and scientific avoidance strategies must be taken seriously. Patients with coronary heart disease who have not been evaluated may experience plaque rupture, acute myocardial infarction or malignant arrhythmias (such as ventricular fibrillation) during intense exercise. Long-term overexertion can lead to an increase in myocardial fibrosis markers, promote pathological myocardial hypertrophy, and at the same time, high-intensity endurance exercise increases the risk of atrial fibrillation (Patel and Link, 2025; Gerardin et al., 2021; Pandey et al., 2015). For patients with absolute contraindications such as unstable angina pectoris, severe aortic stenosis, acute myocarditis/pericarditis, etc., exercise should be strictly restricted. Patients with relative contraindications such as hypertension and severe arrhythmia need to undergo medical assessment before engaging in exercise. When experiencing chest pain/a feeling of oppression, dizziness, arrhythmia or shortness of breath during exercise, one must stop exercising immediately. In conclusion, only through scientific exercise can one maximize the benefits for the heart while avoiding risks.

### 5.3 Telemedicine-enabled cardiac rehabilitation

**FIGURE 2:**
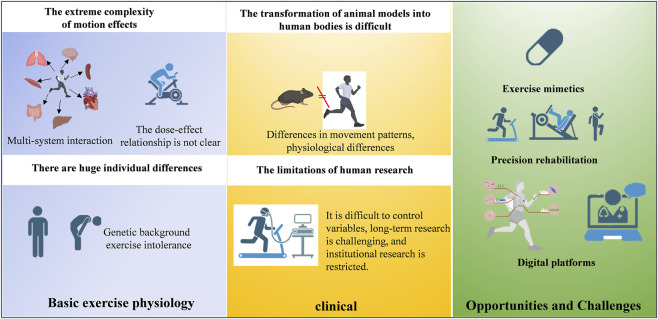
A diagram outlining the translational pipeline from basic exercise physiology to clinical implementation.

Digital transformation is driving a fourth medical revolution in cardiac rehabilitation, featuring wearable biosensors (e.g., Einthoven-style patch ECG), 5G telemedicine platforms, and deep learning-based early warning systems. Studies confirm that Internet of Things (IoT)-enabled home-based cardiac rehabilitation (HBCR) matches center-based programs in reducing major cardiovascular events and improving 6-min walk distance (Anderson et al., 2017; McDonagh et al., 2023). Multimodal data fusion enables real-time monitoring of exercise intensity, myocardial oxygen consumption, and arrhythmias, allowing dynamic prescription optimization. However, safety concerns persist for high-risk populations (Thomas et al., 2019), necessitating biomarker-based pre-event warning systems. Future efforts should integrate three tiers: (1) foundational research on predictive biomarkers; (2) technological innovation in remote monitoring; and (3) clinical implementation strategies. This “trinity” framework may shift cardiac rehabilitation from facility-dependent to intelligent, personalized paradigms (Figure 2).

## 6 Conclusion

Exercise serves as a cornerstone intervention strategy for cardiovascular disease prevention and treatment, with its cardioprotective effects arising from the integration of multidimensional molecular regulatory networks and interorgan synergistic interactions. Although challenges persist in developing exercise mimetics due to target selectivity limitations and systemic complexity, multi-omics analysis of exercise factor networks provides new directions for precision drug design. Concurrently, intelligent upgrades in tele-rehabilitation technologies–particularly the integration of wearable devices with AI-based early warning systems–are facilitating the transition from empirical interventions to data-driven decision-making in cardiac care management.

**FIGURE 3:**
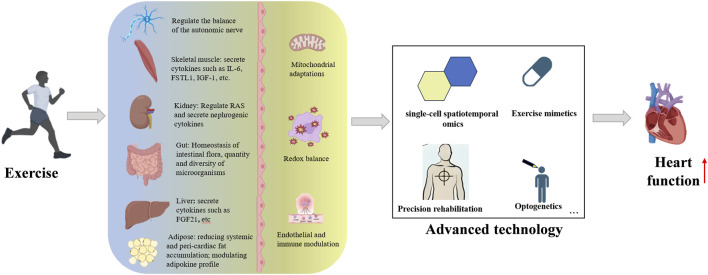
An integrative view of exercise promotes technological progress and heart health.

Current research limitations primarily involve: (1) Insufficient quantitative characterization of dose-effect relationships between exercise parameters and molecular responses; (2) Unclear temporal window characteristics of interorgan communication signals and their interaction mechanisms within pathological microenvironments; (3) Incomplete understanding of maintenance and resolution mechanisms underlying exercise-induced epigenetic memory. Future studies should integrate single-cell spatiotemporal omics, optogenetic modulation, and organoid models to systematically elucidate the hierarchical architecture of exercise-activated cardioprotective networks, thereby establishing theoretical foundations for personalized cardiac rehabilitation strategies. Although the benefits of chronic exercise on physiology and molecular pathways have been established, there is still much to be discovered to establish better-designed clinical protocols and approaches (Figure 3).
